# Harnessing Raman spectroscopy for the analysis of plant diversity

**DOI:** 10.1038/s41598-024-62932-0

**Published:** 2024-06-03

**Authors:** Ekta Jain, Michelle Rose, Praveen Kumar Jayapal, Gajendra P. Singh, Rajeev J. Ram

**Affiliations:** 1 Disruptive and Sustainable Technologies for Agricultural Precision, Singapore-MIT Alliance for Research and Technology, 1 Create Way, 03-06/07/8 Research Wing, Singapore, 138602 Singapore; 2 Research Laboratory of Electronics, Massachusetts Institute of Technology, 77 Massachusetts Avenue, 36-491, Cambridge, MA 02139 USA

**Keywords:** Raman spectroscopy, Biodiversity, Environmental impact, Raman spectroscopy, Plant ecology, Raman spectroscopy

## Abstract

Here, we explore the application of Raman spectroscopy for the assessment of plant biodiversity. Raman spectra from 11 vascular plant species commonly found in forest ecosystems, specifically angiosperms (both monocots and eudicots) and pteridophytes (ferns), were acquired in vivo and in situ using a Raman leaf-clip. We achieved an overall accuracy of 91% for correct classification of a species within a plant group and identified lignin Raman spectral features as a useful discriminator for classification. The results demonstrate the potential of Raman spectroscopy in contributing to plant biodiversity assessment.

## Introduction

Diverse plant ecosystems tend to capture resources more efficiently, cycle nutrients more quickly and are more stable over time^1^. Techniques to rapidly assess plant biodiversity are important as we study and respond to rapid changes in ecosystem biodiversity caused by human development and climate change. Plant spectral diversity has recently emerged as a biodiversity metric that exploits the dissimilarity of leaf-level spectra which capture functional and phylogenetic differences among plant species and can be used as a metric for biodiversity and a measure for critical ecosystem function^1^. To date, spectral diversity measures have focused on variations in hyperspectral (400–2200 nm) reflectance acquired from plant leaves through remote sensing. Advances in imaging systems have led to the development of field-based phenotyping platforms to collect high-throughput trait measurements over a large field of view. With these systems, plants’ physical and physiological features can be interrogated non-destructively. Hyperspectral reflectance on unmanned aerial platforms and by satellite remote sensing allows for scalable plant phenotyping and disease monitoring. Data from these remote platforms can be paired with portable/proximal analytical devices to provide reference data sets free of atmospheric effects, solar illumination, variable orientation due to the three-dimensional structure of the plants^2^. Here, we propose Raman spectroscopy as a tool for proximal sensing of leaf chemistry that can be used to assess biodiversity.

Raman spectroscopy has emerged as a new tool for field-based phenotyping—particularly for early detection of plant stress^3–15^. This involves detecting bacterial infections^12,16^, insect infestations^17^, fungal infections^7,18^, nutrient deficiencies^15,19,20^, and various other pathogens^17,21^. These various stresses manifest through changes in the various metabolites observed in leaf Raman spectra: these include carotenoids, pectin, lignin, carbohydrates such as starch, amino acids, and nitrate.

Farber et al. have used a hand-held Raman spectrometer with partial least square discriminant analysis (PLS-DA) to distinguish poison ivy from weeds, grasses, and trees^22^. Here, we extend this work to a wider range of vascular plants with a specific focus on diverse classes and perform the survey of Raman spectra across multiple plant species with the goal of identifying differences between diverse plant species *and classes*. The primary plant groups in the plant kingdom include Bryophytes, Pteridophytes, Gymnosperms, and Angiosperms^23–26^. In this study, we focused on vascular plant groups commonly found in forest ecosystems, specifically angiosperms (both monocots and eudicots) and pteridophytes (ferns).

## Spectral collection and analysis

**Figure 1:**
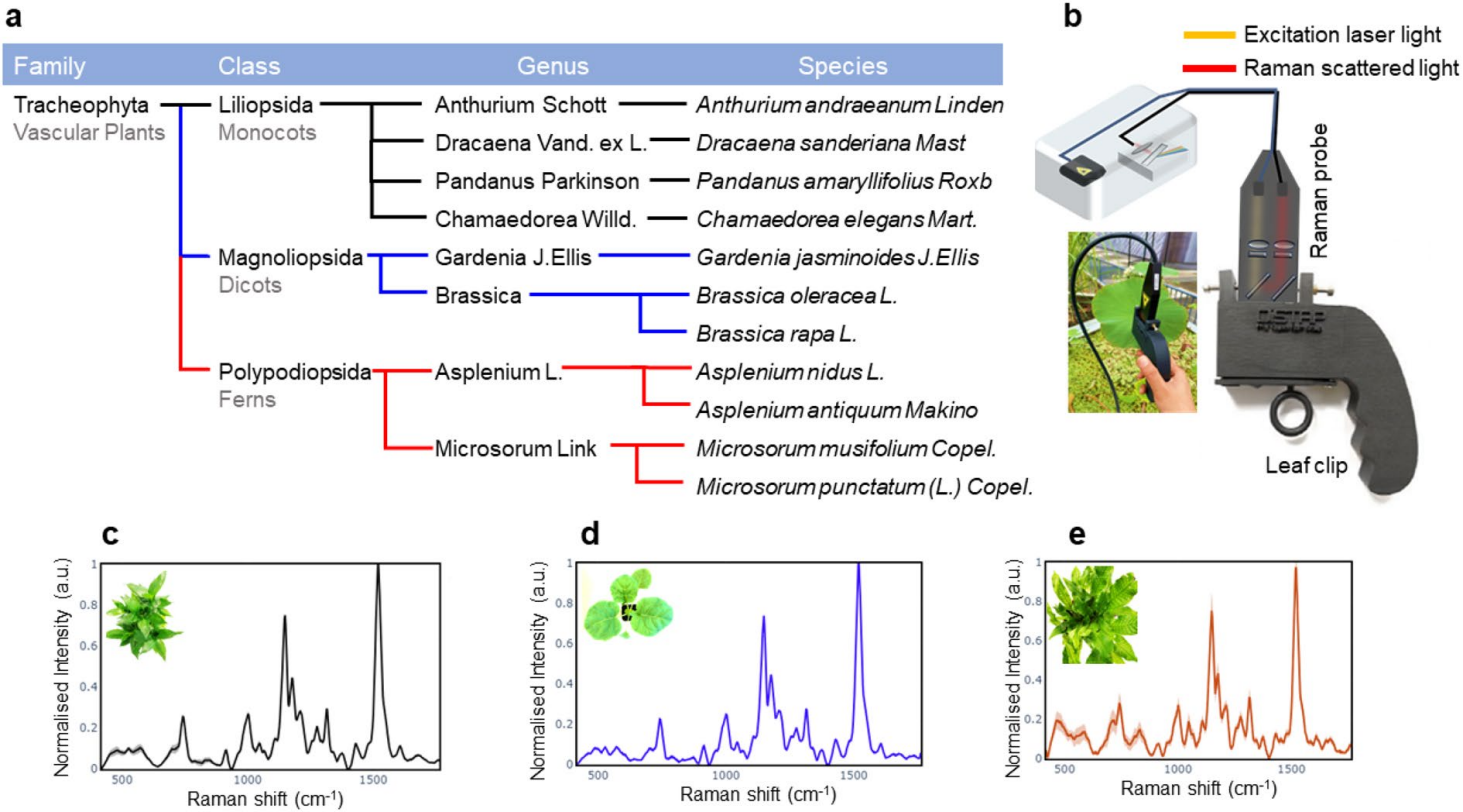
(**a**) The classification of vascular plants into major classes within the plant kingdom. The classification is based on various criteria such as morphological features, reproductive structures, evolutionary history, and genetic relationships. The major classes typically include Liliopsida, Magnoliopsida, and Polypodiopsida. The study focuses on the Raman analysis of ferns (polypodiopsida), along with monocots (liliopsida) and eudicots (magnoliopsida). (**b**) A mobile leaf clip Raman sensor used in this study for outdoor plants under full light conditions^15^. (**c**) A 9-week-old ‘lucky bamboo’ monocot showcasing its Raman spectra (black spectra). (**d**) ‘Kai Lan’ eudicot at 3 weeks of age and the associated spectra (blue spectra). (**e**) 10-week-old ‘crocodile’ fern alongside its corresponding Raman spectra (red spectra). Raman spectra were collected from five distinct leaves, with two measurements taken at separate locations on each leaf. The resulting spectra include a total of ten measurements for each type of plant, represented in light colours (red, black, blue), while the corresponding averages are depicted in darker shades (red, black, blue). All Raman measurements were conducted at a laser power of 130 mW with a 10 s acquisition time.

In this work, we have made use of the portable, flexible, and field-friendly nature of portable Raman spectroscopy for on-site plant identification in various environments, including, urban farms/gardens or agricultural fields. The leaf clip Raman sensor used here consists of a Raman Fiber probe connected to a portable Raman instrument using an 830 nm excitation laser^15^. The laser power was adjusted to 130 mW to minimize sample damage and fluorescence interference. The laser system (830 nm excitation, Innovative Photonic Systems USA) and spectrometer setup (Avantes HSC Symmetrical Czerny-Turner) were combined within a compact, portable instrument enclosure, which featured software controls, as developed by TechnoSpex Pte Ltd (as depicted in Fig. 1b^15^). This integrated instrument covered a spectral range extending from 100 to 2000 cm^−1^, achieving a spectral resolution of 10 cm^−1^.

The Raman leaf-clip sensor was utilised for in vivo and in situ acquisition of Raman spectra under indoor growing conditions for three different types of plants: ferns, eudicots, and monocots. Raman spectra were collected on two locations (left and right side)^13^ for five leaves on each individual plant with an integration time of 10 s. At each location, five Raman spectra were acquired on the adaxial side of the leaf. The cosmic ray interference was detected and eliminated within the collected spectra. After the cosmic ray removal, a smoothing procedure was applied to the individual spectra across their respective wavelength domains using the Savitzky–Golay filter function^27,28^, a well-established method for spectral noise reduction. A representative sample spectrum was obtained by computing the mean values of the five filtered and smoothed spectra at each wavelength. To generate the Raman spectrum showcased in the results section, remaining fluorescence artifacts were effectively removed using a positive residual-style polynomial subtraction technique^27^. All these data processing procedures were implemented within the MATLAB and the calibration of the Raman shift was validated through polystyrene spectra (a well-known and widely documented Raman spectrum^29^).

For the Raman analysis, plants ranging from 3 to 15 weeks’ old grown in soil were chosen, which have different leaf shapes, sizes, and textures. Four Monocots namely, *Anthurium andraeanum* (flamingo lily), *Dracaena sanderiana* (lucky bamboo), *Pandanus amaryllifolius* (pandan), and *Chamaedorea elegans* (parlour palm); Three Eudicots: *Gardenia jasminoides* (cape jasmine), *Brassica oleracea* (Kai Lan), and *Brassica rapa* (Pak Choi); and Four Ferns: *Asplenium nidus* (bird’s nest), *Microsorum musifolium* (crocodile), *Asplenium antiquum* (Osaka) and *Microsorum punctatum* (fish-tail). All plants used in this study had three biological replicates and have been purchased from nursery (Candy Floriculture Pte Ltd) except the two Eudicots (Kai Lan and Pak Choi) that were grown in our Disruptive and Sustainable Technologies for Agricultural Precision (DiSTAP) Lab. The plants were not at the same stage of vegetation. Three biological replicates of cape jasmine plants were in the seed stage, while three biological replicates of flamingo lily were in the flowering stage. Overall, we have used 12 Monocots, 9 Eudicots and 12 Ferns for the Raman measurements. Figure 1a, c–e shows the plant kingdom classification and different species used in this study for Raman measurements along with their Raman spectra.

### Statement for research involving plants

Plants were procured from a nursery, and few plants were cultivated in our laboratory. All experimental procedures adhered to the relevant guidelines and regulations.

**Figure 2:**
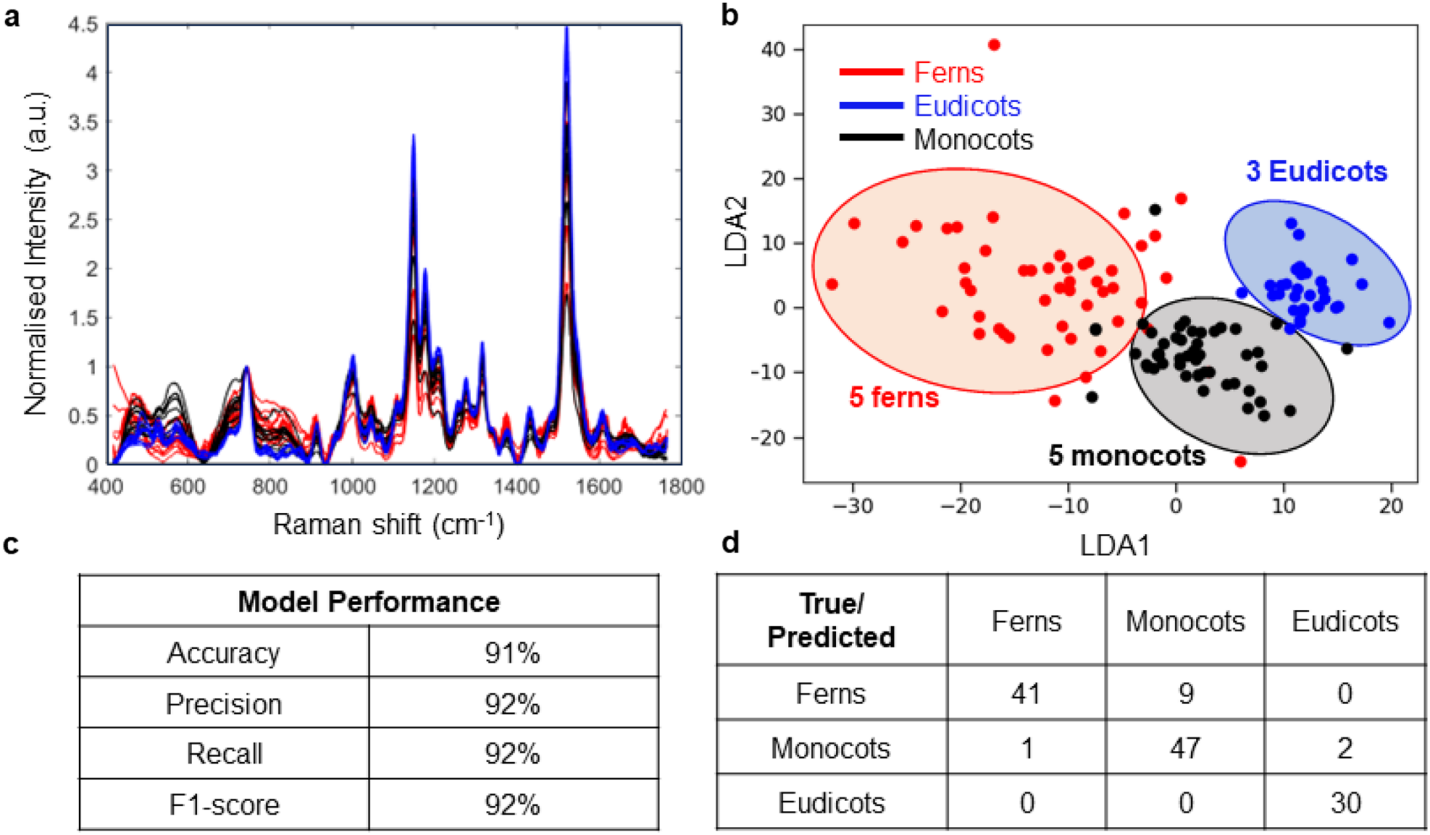
This figure shows the application of logistic regression on the test set within the context of Linear Discriminant Analysis (LDA) when entire Raman spectra range (400–1750 cm^−1^) is selected. (**a**) Raman spectra of 12 ferns, 12 monocots and 9 eudicots for both test and training sets. (**b**) The test set plot showcases the predictive modelling and evaluation of the test set using logistic regression, providing insights into the classification performance and accuracy of the LDA model. 5 ferns, 5 monocots, and 3 eudicots have been used for the test set. (**c**) The performance evaluation of the Linear Discriminant Analysis (LDA) model, showcasing accuracy, precision, recall determining its effectiveness in distinguishing different plant species. LDA successfully distinguishes among the three distinct plant types, with an accuracy of 91% in the test set and 100% accuracy in the training set. (**d**) The confusion matrix for the linear discriminant analysis (LDA) model provides a detailed breakdown of true positive, true negative, false positive, and false negative classifications, offering a complete assessment of the model's predictive performance for distinguishing plant species.

To further explore the distinctions between plant species based on spectral features, we employed linear discriminant analysis (LDA), a statistical technique widely applied for pattern recognition and classification^30,31^. This involved identifying plant species based on their characteristic attributes. We used Scikit-learn—a machine learning module in Python for performing LDA^32,33^. For LDA model training, we utilized a dataset comprising 7 ferns, 7 monocots, and 6 eudicots. For the test set, we employed 5 ferns, 5 monocots, and 3 eudicots. Figure 2a shows the Raman spectra of the entire dataset i.e. 12 ferns (3 biological replicates with 4 different species), 12 monocots (3 biological replicates with 4 different species) and 9 eudicots (3 biological replicates with 3 different species). Analyzing the Raman spectra across all classes presented challenges in identifying notable variations in peak intensities. This implies that distinguishing peak variability among classes is difficult, and the collective contribution of each spectral segment contributes to the overall accuracy of the LDA model. Each plant class consists of 1 spectrum, and each spectrum presents the Raman scattering specific to the respective plant species, offering valuable insights into their molecular composition and structural features. Figure 2b displays the LDA logistic regression model for the test set across the complete spectral range (400–1750 cm^−1^) with respect to the two discriminant functions, LDA1 and LDA2. Notably, LDA effectively distinguishes among the three distinct plant types, achieving an accuracy of 91% in the test set and a perfect 100% accuracy in the training set. The proximity of the monocots and eudicots in Fig. 2b suggests they are part of the same group of angiosperms. Figure 2c provides an overview of the performance metrics employed to evaluate the LDA model’s effectiveness in plant identification, including accuracy, precision, recall and f1-score for the test set. Furthermore, Fig. 2d presents the confusion matrix, displaying the count of true positives, true negatives, false positives, and false negatives for each plant class, offering a comprehensive understanding of the model's performance in plant identification. The confusion matrix illustrates a minor misclassification between ferns and monocots (9 misclassifications), between monocots and eudicots (2 misclassifications), and between eudicots and ferns (0 misclassifications).

**Figure 3:**
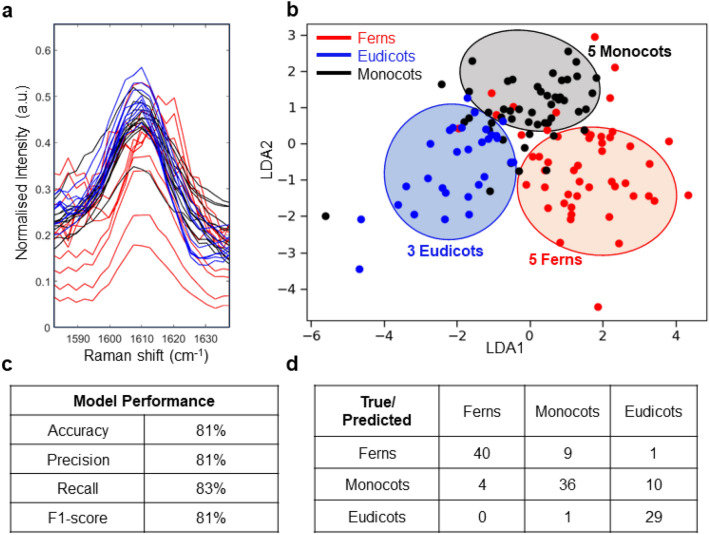
(**a**) Enlarged lignin peak region (1580–1630 cm^−1^) for all plant species: red for ferns, black for monocots and blue for eudicots. (**b**) The logistic regression plot of the test set providing insights into the classification performance and accuracy of the LDA model. (**c**) The performance evaluation of the LDA model. LDA distinguishes among the three distinct plant types, with an accuracy of 81% in the test set when only the lignin region is considered. (**d**) The confusion matrix for the LDA model for the lignin region.

To identify the class of molecules that provide the clearest discrimination between plant classes, we have repeated the classifier construction using spectral ranges restricted to individual peaks. Figure 3a displays the Raman peaks within the lignin region for ferns (in red), monocots (in black), and eudicots (in blue). Within the classification of angiosperms, both eudicots and monocots exhibit similar lignin compositions, whereas ferns lack the *p*-hydroxyphenyl component. Thus, we observed minimal differences in lignin peak intensities between eudicots and monocots when contrasted with those observed in ferns. A discussion on the lignin composition in angiosperms (monocots and eudicots) and ferns can be found in the results section of this manuscript. Figure 3b shows plots of the LDA logistic regression model specifically to the lignin region (1580–1630 cm^−1^) to understand the impact of the lignin peak in distinguishing the three classes. Significantly, the LDA model effectively distinguishes among the three distinct plant types, attaining an accuracy of 81% in the test set and 81.2% accuracy in the training set (Fig. 3b). The decrease in accuracy suggests that the model performs better when considering the entire spectral range; however, the lignin region indeed plays a substantial role in distinguishing different classes over other peaks. Figure 3c provides performance metrics for the lignin region, assessing the LDA model’s efficacy in plant identification, including accuracy, precision, recall, and f1-score for the test set; and Fig. 3d presents the confusion matrix, describing the count of true positives, true negatives, false positives, and false negatives for each plant class.

### Statement of consent

The informed consent was obtained from the participant researcher for publication of photograph in the supplementary figure (Fig. S2).

## Results and discussion

The Raman leaf clip sensor was able to acquire Raman spectra from all the different types of plant leaves. Figure 2a shows the Raman spectra that resulted from different plant classes and the Raman spectra shows several common Raman peaks. Based on Raman spectrum of carotenoid chemical standards, we identified the 1525 cm^−1^, 1155 cm^−1^ and 1000 cm^−1^ peaks to be present in all plant species samples (Table 1). The observed Raman peak at 1525 cm^−1^ is due to a C=C stretching vibration (ν_1_), the 1155 cm^−1^ peak is a C–C stretching vibration (ν_2_), and the weak peak at 1000 cm^−1^ is due to C–CH_3_ stretching (ν_3_)^34^. The observed Raman peak at 1604 cm^−1^ is associated with the stretching vibrations of the aromatic rings in lignin, which is a characteristic feature of lignin’s structure^35–38^. The other peaks associated with lignin in Raman spectroscopy are typically found in the following regions: 1265–1326 cm^−1^ (guaiacyl ring breathing and C–C stretching); Peaks in this region are related to the guaiacyl ring structure present in lignin^39–41^. The region between 1440 and 1488 cm^−1^ is associated with bending and deformation vibrations of C–H bonds in lignin^39,40^.

**Table 1:** Vibrational bands and their corresponding assignments for plant leaves.

Band (Raman peak cm^−1^)	Vibrational mode	Assignment
520	ν(C–O–C) glycosidic	Cellulose^42^
747	γ(C–O–H) of COOH	Pectin^43^
1000	C–CH_3_ stretching (ν_3_) and phenylalanine	Carotenoids, proteins^44,45^
1155	C–C stretching, asymmetric ring breathing	Carotenoids^46^, cellulose^47^
1265	Guaiacyl ring breathing, C–O stretching (aromatic)	Phenylpropanoids^48^
1326	δCH_2_ bending vibration	Cellulose, phenylpropanoids^42^
1440	δ(CH_2_) + δ(CH_3_)	Aliphatic^49^
1488	δCH_2_ bending vibration	Aliphatic^49^
1525	C=C stretching vibration (ν_1_)	Carotenoids^50^
1604	ν(C–C) aromatic ring	Lignin^35,36^

Lignin is a complex and rigid polymer that plays a critical role in the structural support and rigidity of plant cell walls^36,51^. It is a major component of the secondary cell walls in vascular plants, including both ferns (non-flowering plants) and angiosperms (flowering plants). The composition and structure of lignin can vary between different plant species and even within different parts of the same plant. The lignin composition in ferns mainly consists of guaiacyl (G) and syringyl (S) monolignols, with guaiacyl being the dominant type^52–54^. Angiosperms have a more complex lignin composition compared to ferns. Lignin in angiosperms contains three main types of monolignols: guaiacyl (G), syringyl (S), and *p*-hydroxyphenyl (H)^51,55^. The proportion and arrangement of these monolignols can vary between different plant tissues and species^51,55^. For example, hardwoods primarily contain G and S lignin, while softwoods mainly consist of G lignin^52^. The composition of lignin in angiosperms can also vary depending on the developmental stage of the plant, the specific tissue (e.g., xylem, phloem), and environmental factors^38,52^.

## Conclusion

This study demonstrates the classification of plant species using Raman spectroscopy. It showcases the potential of Raman spectroscopy, when coupled with appropriate data analysis and classification techniques such as LDA, can be a powerful species-independent analytical tool for accurate and rapid identification of plant species based on their unique spectral fingerprint obtained from Raman scattering. The utilization of Linear Discriminant Analysis (LDA) in combination with a comprehensive dataset of Raman spectra enabled a detailed exploration of the unique features characterizing different plant species. The LDA plot presented in Fig. 2b, depicts a clear separation among ferns, monocots, and eudicots based on their spectral attributes. This visual representation highlights the potential of LDA as a powerful tool for precise plant species identification, essential for a wide array of applications in the realm of botanical research and beyond. Moreover, the LDA model achieved a high accuracy of 91%, indicating its effectiveness in distinguishing plant species based on the lignin peak. It shows a well-defined separation of the plant species, suggesting the efficacy of the selected features (particularly lignin) for plant identification using LDA.

As demonstrated here, Raman spectroscopy is a truly species agnostic tool for assessment of leaf chemical composition. The extension of this initial work to stand-off Raman spectroscopy with a large field-of-view would allow for the rapid classification of plant species and the assessment of plant biodiversity in a variety of ecosystems. Such a chemically-specific, optical instrument would complement existing tools and protocols for the assessment of ecosystem biodiversity.

### Supplementary Information

Supplementary Figures.

## Data Availability

The datasets generated during and/or analysed during the current study are available from the corresponding author upon reasonable request.
