# Supplementary material for: Harnessing Raman spectroscopy for the analysis of plant diversity
Source: Sci Rep. 2024 Jun 3;14:12692. doi: 10.1038/s41598-024-62932-0 (PMC11148151; doi:10.1038/s41598-024-62932-0)
Supplement: Supplementary file 1 — Supplementary Figures. [file 41598_2024_62932_MOESM1_ESM.pdf]

Supplementary Figures

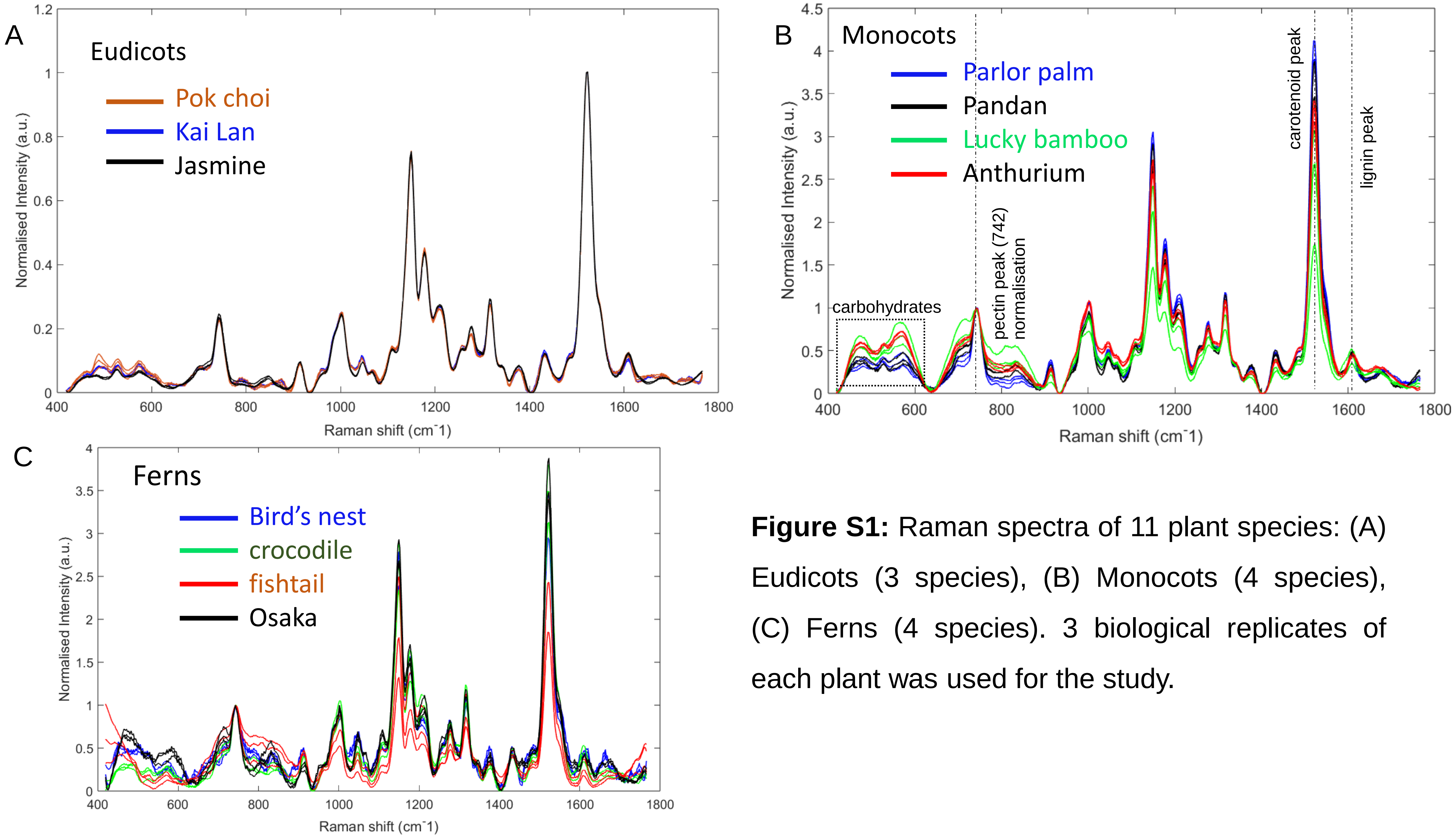

Figure S1: Raman spectra of 11 plant species: (A) Eudicots (3 species), (B) Monocots (4 species), (C) Ferns (4 species). 3 biological replicates of each plant was used for the study.

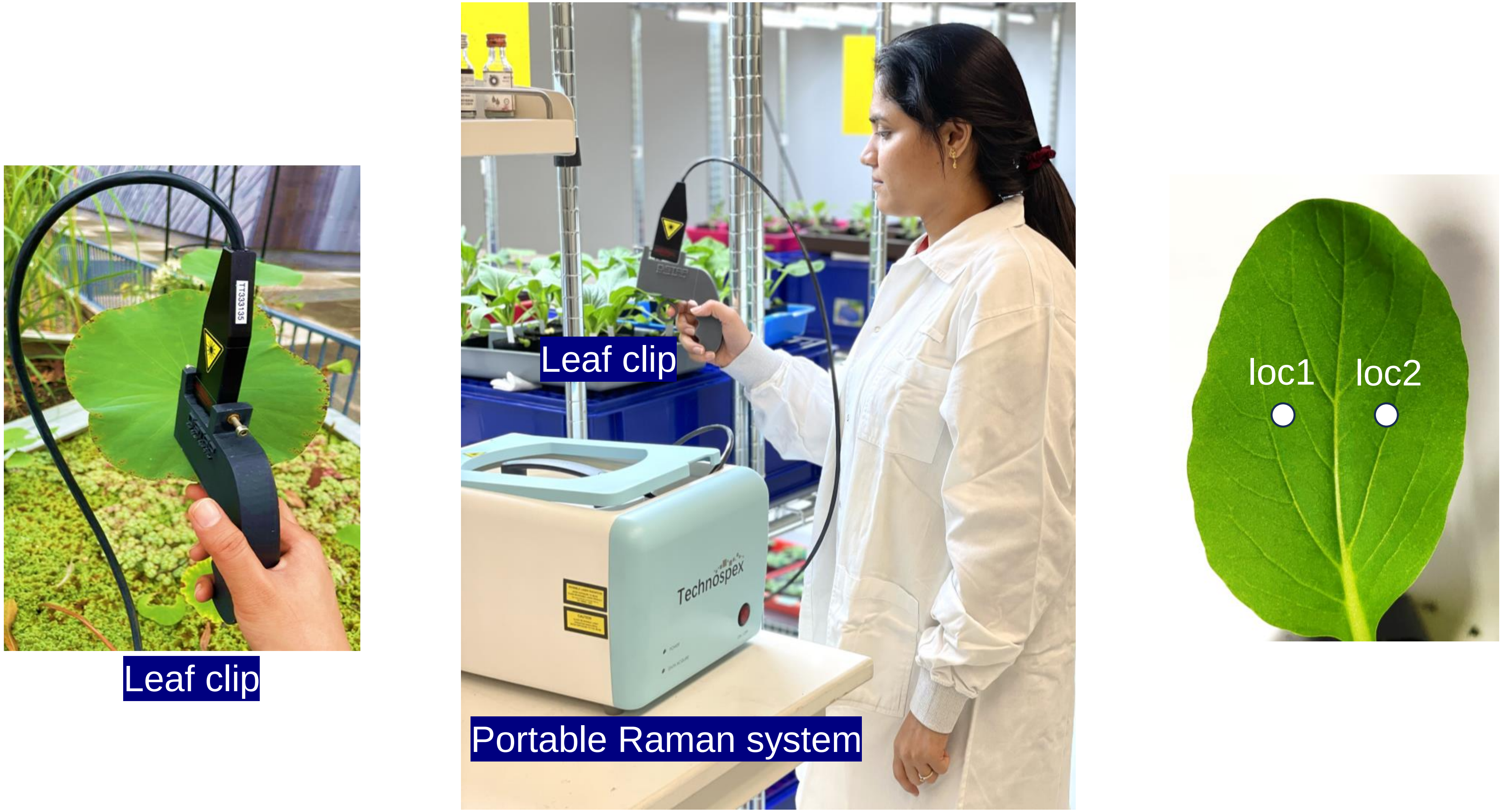

Figure S2: Photo of portable Raman spectroscopy system (with leaf clip) that was used to acquire Raman spectra at 830 nm laser excitation. Raman spectra was taken at 2 locations (loc1 and loc2) on each leaf, 5 different leaves of each plant was considered.

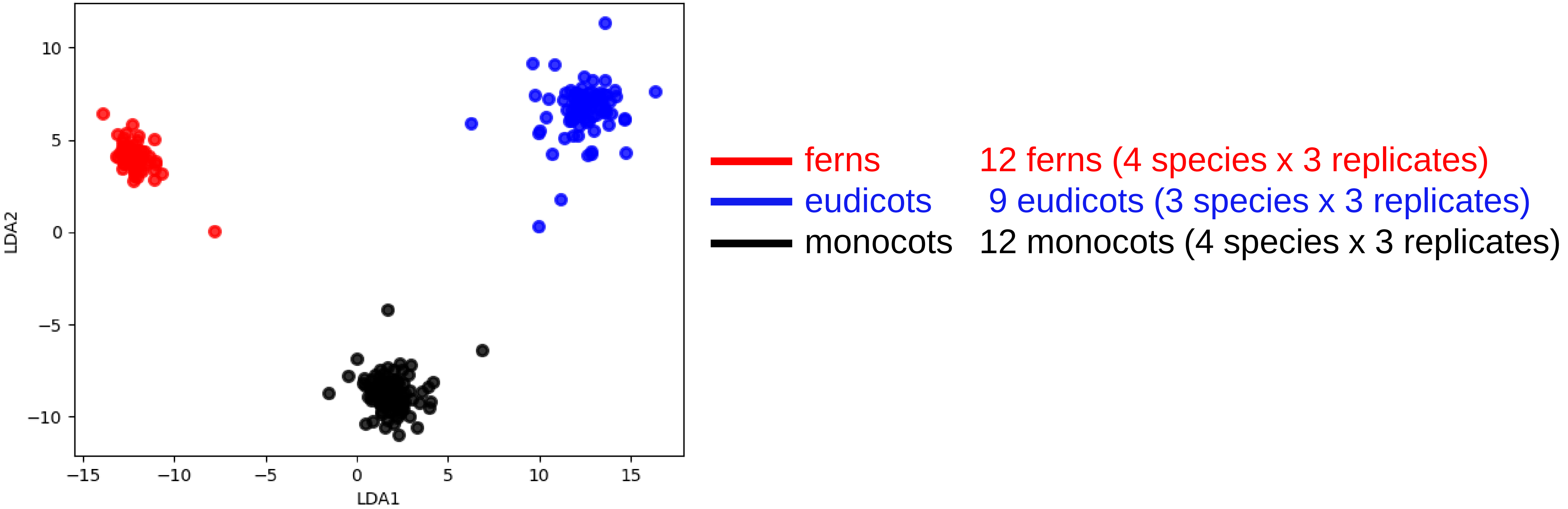

Figure S3: LDA scatter plot shows the effective discrimination and separation of plant species (Ferns in red, Monocots in black and Eudicots in blue) based on distinct features observed in the Raman spectra. The plot provides a visual representation of the discriminant power of the selected features in distinguishing between different plant groups using LDA. 4 species of ferns, 3 species of eudicots and 4 species of monocots, each with 3 biological replicates were utilized in this study.

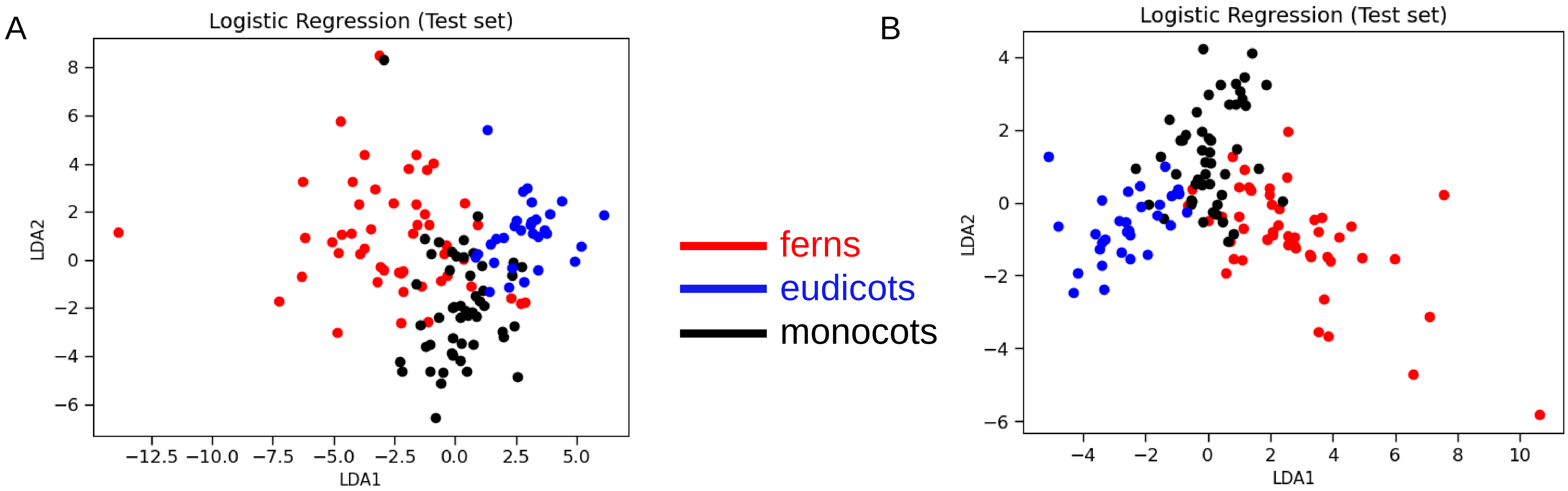

Accuracy 78.5% :
Carbohydrates (400-600 cm^{-1})
region in the Raman spectra

Accuracy 77.69% :
Carotenoid (1480-1560 cm^{-1})
region in the Raman spectra

Figure S4: This figure shows the application of logistic regression on the test set within the context of Linear Discriminant Analysis (LDA) when only **(A)** Carbohydrates region is considered **(B)** Carotenoid region is considered. The test set plot showcases the predictive modelling and evaluation of the test set using logistic regression. Species of 5 ferns, 5 monocots, and 3 eudicots have been used for the test set.

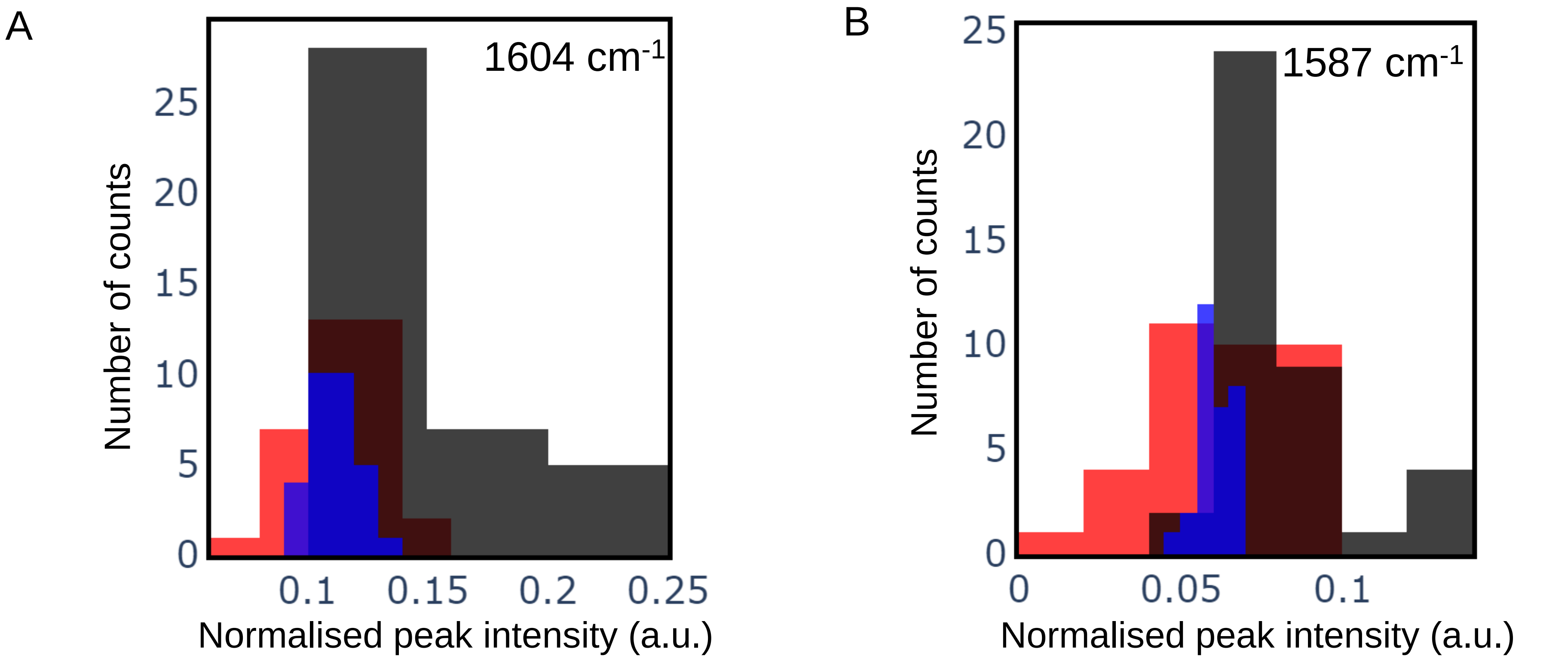

Figure S5: This figure shows the histogram plot of three classes: ferns (red), monocots (black) and eudicots (blue), focusing on the (A) Lignin peak intensity (1604 cm^{-1}) alongside (B) off peak (1587 cm^{-1}). The lignin peak (1604 cm^{-1}) intensity shows minimal differences in intensities between eudicots and monocots when contrasted with those observed in ferns. Furthermore, the shift in peak intensities of ferns, as seen in (B), suggests that the lignin peak intensities among the three classes assist to differentiate ferns from the rest of the spectra. This study used species of 4 ferns, 4 monocots, and 3 eudicots, with three biological replicates for each plant. Spectra were taken from five distinct leaves of each plant, at two separate locations.
